# Targeted enzyme prodrug therapy for metastatic prostate cancer – a comparative study of L-methioninase, purine nucleoside phosphorylase, and cytosine deaminase

**DOI:** 10.1186/s12929-014-0065-3

**Published:** 2014-07-22

**Authors:** Katrin P Guillen, Carla Kurkjian, Roger G Harrison

**Affiliations:** 1 Bioengineering Center and the School of Chemical, Biological and Materials Engineering, University of Oklahoma, Norman, OK, USA; 2 Oncology-Hematology Section, Health Sciences Center, University of Oklahoma, Oklahoma City, OK, USA; 3 Stephenson Cancer Center, Health Sciences Center, University of Oklahoma, Oklahoma City, OK, USA

**Keywords:** Enzyme prodrug therapy, Vascular-targeted, Docetaxel, Annexin V, Prostate cancer

## Abstract

**Background:**

Enzyme prodrug therapy shows promise for the treatment of solid tumors, but current approaches lack effective/safe delivery strategies. To address this, we previously developed three enzyme-containing fusion proteins targeted via annexin V to phosphatidylserine exposed on the tumor vasculature and tumor cells, using the enzymes L-methioninase, purine nucleoside phosphorylase, or cytosine deaminase. In enzyme prodrug therapy, the fusion protein is allowed to bind to the tumor before a nontoxic drug precursor, a prodrug, is introduced. Upon interaction of the prodrug with the bound enzyme, an anticancer compound is formed, but only in the direct vicinity of the tumor, thereby mitigating the risk of side effects while creating high intratumoral drug concentrations. The applicability of these enzyme prodrug systems to treating prostate cancer has remained unexplored. Additionally, target availability may increase with the addition of low dose docetaxel treatment to the enzyme prodrug treatment, but this effect has not been previously investigated. To this end, we examined the binding strength and the cytotoxic efficacy (with and without docetaxel treatment) of these enzyme prodrug systems on the human prostate cancer cell line PC-3.

**Results:**

All three fusion proteins exhibited strong binding; dissociation constants were 0.572 nM for L-methioninase-annexin V (MT-AV), 0.406 nM for purine nucleoside phosphorylase-annexin V (PNP-AV), and 0.061 nM for cytosine deaminase-annexin V (CD-AV). MT-AV produced up to 99% cell death (*p* < 0.001) with limited cytotoxicity of the prodrug alone. PNP-AV with docetaxel created up to 78% cell death (*p* < 0.001) with no cytotoxicity of the prodrug alone. CD-AV with docetaxel displayed up to 60% cell death (*p* < 0.001) with no cytotoxicity of the prodrug alone. Docetaxel treatment created significant increases in cytotoxicity for PNP-AV and CD-AV.

**Conclusions:**

Strong binding of fusion proteins to the prostate cancer cells and effective cell killing suggest that the enzyme prodrug systems with MT-AV and PNP-AV may be effective treatment options. Additionally, low-dose docetaxel treatment was found to increase the cytotoxic effect of the annexin V-targeted therapeutics for the PNP-AV and CD-AV systems.

## Background

Prostate cancer (PC) is the most common non-skin malignancy and the second leading cause of cancer-related death in American men [1], yet remains essentially incurable. Since the introduction of PSA specific screening, the lethality of prostate cancer stems not from a lack of early detection but more commonly from the failure of loco-regional therapies creating a need for improved systemic therapies [2]. Currently, most single-agent anticancer drugs face challenges due to increased multidrug resistance [3], pharmacokinetic limitations [4],[5], and restricted clinical dosage or frequency of administration due to cytotoxicity in non-cancerous tissues [6]–[8].

Antibody-directed enzyme prodrug therapy (ADEPT), gene-directed enzyme prodrug therapy (GDEPT), and viral-directed enzyme prodrug therapy (VDEPT) have been investigated as means to utilize enzymes to convert relatively non-toxic prodrugs into clinically relevant concentrations of cytotoxic drugs directly at tumor sites. However, all three of these approaches have significant limitations [9]–[11]. To improve upon the clinical applicability, efficacy, and safety of enzyme prodrug therapy, we previously developed three fusion proteins (FPs), each targeted to primary tumors, their metastases, and the tumor vasculature. This dual targeting strategy allows for two distinct mechanisms of killing: (i) via the direct action of the cytotoxic drug on the tumor cells, and (ii) by killing tumor vasculature endothelial cells and thereby effectively cutting off the tumor blood supply. Vascular targeting makes these FPs an attractive option because endothelial cells are relatively genetically-stable, easily-accessible targets that enable therapeutic effect amplification through tumor infarction, as well as tumor-type independent targeting [12]. PC is especially well suited to this dual targeting strategy as prostate carcinomas have been shown to have approximately twice the vascular density of healthy prostate tissue [13] and microvessel density serves as a predictor of cancer-specific survival [14]. To date, the efficacy of these targeted enzyme prodrug systems on PC has remained unexplored.

Human annexin V (AV) is used to target each FP. AV has a strong affinity to the anionic phospholipid phosphatidylserine (PS), normally tightly segregated to the inner leaflet in eukaryotic plasma membranes [15], but robustly and consistently expressed on the outer leaflet in a wide range of cancer cell lines, their metastases [16],[17], and the luminal side of tumor endothelium [18],[19]. To maximize FP binding to tumor cells, we investigated treatment with docetaxel, a tubulin/microtubule targeting chemotherapeutic agent [20], which is becoming increasingly important in combination therapies for metastatic, hormone-refractory PC [21]. Therapeutic docetaxel dosage is limited by drug toxicity [7] but a single subtoxic dose has been shown to increase PS exposure on tumor endothelium by ~70% without causing apoptosis or changing PS exposure on normal endothelium [22]. This large increase in AV binding sites has the potential to increase the cytotoxic power of our enzyme prodrug systems.

We previously developed three AV-targeted FPs, each containing a non-human enzyme [23]–[25]. The enzymes utilized are:

(i) L-methioninase (MT), which converts L-selenomethionine (SeMet) to toxic methylselenol, α-ketobutyrate, and ammonia [26]. MT also converts the amino acid methionine to methanethiol, which provides a second point of attack since most cancer cells exhibit increased methionine-dependence [27],[28]. PC cell lines have shown sensitivity to non-targeted MT/SeMet treatment *in vitro*[29].

(ii) Purine nucleoside phosphorylase (PNP), which converts fludarabine (FD) into highly cytotoxic 2-fluoroadenine (2-FA) that incorporates into DNA/RNA, thereby effectively killing both dividing and nondividing cells [30]. PNP exhibits a powerful bystander effect [31],[32], and PC cells have shown sensitivity to PNP/FD GDEPT treatment [33]–[35].

(iii) Cytosine deaminase (CD), which converts the nucleoside analog 5-fluorocytosine (5-FC) to the more toxic pyrimidine analog 5-fluorouracil (5-FU), metabolites of which misincorporate into DNA/RNA and inhibit the nucleotide synthesis enzyme thymidylate synthetase [36]. PC cell lines have shown sensitivity to 5-FU and GDEPT CD/5-FC treatment [37].

To address the vascular targeting capabilities of these enzyme prodrug systems, we have previously shown that all three FPs bind tightly to PS expressing human abdominal aorta endothelial cells (HAAE-1) *in vitro*, with dissociation constants ranging from 0.5-1.5 nM [23]–[25]. Cytotoxic efficacy of our FP systems on HAAE-1 cells has also been demonstrated previously *in vitro,* with cell killing ranging from 5-100% [23]–[25]. We have validated these *in vitro* methods for determining vascular targeting/cytotoxic efficacy via the successful transition of the MT-AV/SeMet system *in vivo* for mice with implanted MDA-MB-231 breast tumors [38].

In the present study, we characterize the binding and evaluate the *in vitro* anticancer efficacy of three enzyme prodrug systems on PC-3 human prostate carcinoma cells in the presence and absence of low-dose docetaxel treatment.

## Methods

### Expression and purification of fusion proteins

All FPs were expressed and purified as described previously [23]–[25]. Briefly, polymerase chain reaction (PCR) was used amplify genes encoding each enzyme, a six residue flexible linker, annexin V, an N- or C- terminal His_6_ tag, and an engineered HRV 3C protease cleavage site. Plasmids containing each FP were created via transformation of NovaBlue competent cells and then expressed in *E.coli* BL21 (DE3) cells. Recombinant FPs were produced and purified according to the procedure of Zang *et al.*[39] using immobilized metal (Ni^2+^) affinity chromatography. The His_6_ tag was removed during purification by cleavage with HRV-3C protease (Merck, Darmstadt, Germany). FPs were lyophilized and stored at −80°C.

### Cell culture

The PC-3 human prostate adenocarcinoma cell line was obtained from the American Type Culture Collection (ATTC, Manassas, VA, USA) and cultured in F-12 K medium (ATTC) supplemented with 10% fetal bovine serum, 100 U/ml penicillin, and 100 μg/ml streptomycin (all from Atlanta Biologics, Flowery Branch, GA, USA) at 37°C in a 5% CO_2_ atmosphere. Cells were passaged at 70-80% confluence, 2–3 times per week, less than 12 times during the course of experiments.

### *In vitro* binding assays

Cells were grown in T-75 flasks to 70-80% confluence, plated at 50 k cells/well in 24-well cell culture plates, and allowed to grow to 90% confluence. Dissociation constants were determined as described previously [23]–[25]. Briefly, cells were fixed with 0.25% glutaraldehyde in PBS, then quenched with 50 nM NH_4_Cl in PBS. After a 1 h of incubation with 0.5% BSA in PBS, cells were washed, and varying concentrations (0–20 nM) of SureLINK biotin (KPL, Gaithersburg, MD, USA) labeled FPs were added and allowed to bind at 37°C for 2 h. Cells were washed with PBS containing 0.5% BSA and treated with streptavidin-horseradish peroxidase (2 μg/ml, KPL) for 1 h at room temperature. Cells were washed, and HRP was quantified via chromogenic substrate *o*-phenylenediamine (0.4 mg/ml) in 0.05 mM phosphate–citrate buffer (pH 5.0) containing 0.012% hydrogen peroxide. Since Ca^2+^ is essential for AV binding to PS, the above procedure was conducted in the presence of 2 mM Ca^2+^ (total binding) and in the absence of Ca^2+^ with 5 mM EDTA to chelate any residual Ca^2+^ (non-specific binding). All experiments contained a blank subjected to the same procedure but with 0 nM FP.

### *In vitro* enzyme prodrug cytotoxic efficacy

Studies were carried out over a 6-day (MT-AV, PNP-AV) or 9-day (CD-AV) treatment cycle. Cells were plated as described previously, but only allowed to reach 50-60% confluence. Prior to the first viability assay, selected wells were pre-treated with 50 pM docetaxel (Biotang, Waltham, MA, USA). All medium was enhanced with 2 mM Ca^2+^ since annexin V binding is calcium-dependent. Medium for MT-AV cytotoxicity studies was also supplemented with 0.02 mM pyridoxal phosphate (co-factor). Cells were treated with a saturating concentration of FP (100 nM) every 3 days for 2 h at 37°C in accordance with previous binding stability studies [23]–[25]. Each day, medium was replaced with medium containing varying concentrations of prodrug (L-SeMet and 5-FC from Fisher Scientific, Waltham, MA, USA and FD from VWR, Radnor, PA, USA) or drug analog (2-FA from Fisher Scientific and 5-FU from Sigma-Aldrich, St. Louis, MO, USA) with or without 50 pM docetaxel. This docetaxel concentration was chosen since it is similar to the level previously reported to lead to PS exposure *in vitro* without a cytotoxic effect on cells [22]. An Alamar Blue (Invitrogen, Grand Island, NY, USA) assay was preformed every 2–3 days to measure cell viability [40]. Cells were incubated with 10% Alamar Blue in fresh media for 4 h at 37°C. From each well, 250 μl was transferred to an opaque 96-well plate, and fluorescence (530/590 nm) was read on a microtiter plate reader. Cells were washed twice after each Alamar Blue assay and three times after each FP incubation before prodrug/drug treatments were added.

### Data analysis

All treatments were run in triplicate. Dissociation constants were obtained using Prism 5 software (GraphPad, La Jolla, CA, USA). Statistical significance was determined with Prism 5 via a one-way ANOVA employing the Tukey-Kramer multiple comparisons test.

## Results

### Binding strength

**Figure 1:**
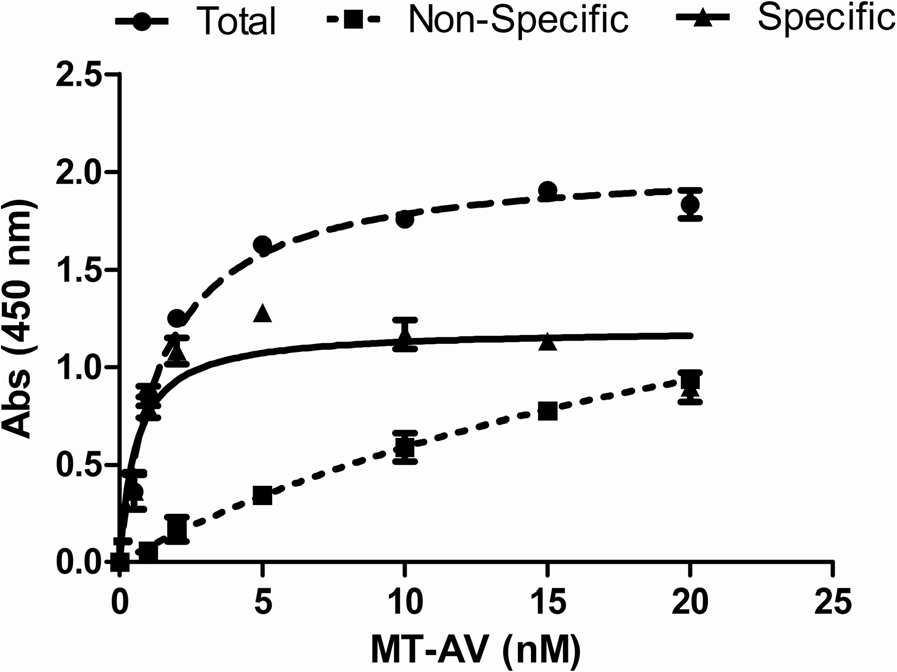
**Binding strength of MT-AV to PC-3 cell surface PS.** PC-3 cells were incubated with increasing concentrations of biotin labeled MT-AV with total binding (●) measured in the presence of 2 mM Ca^2+^ and non-specific binding (■) measured in the absence of Ca^2+^ with 5 mM EDTA to chelate any residual Ca^2+^. Specific binding (▲) was obtained by subtracting non-specific from total binding. Data presented as mean ± SE (n = 3).

The ability of each FP to bind to PS on the PC-3 cell surface was determined by measuring the total binding and non-specific binding and subtracting to obtain specific binding, with typical results shown in Figure 1 for MT-AV. The dissociation constant (K_d_) was calculated utilizing a one-site, non-competitive binding model, and K_d_ values are presented in Table 1.

**Table 1:** **Dissociation constant (K**_**d**_**) of each fusion protein binding to PC-3 cells**

**Fusion protein**	**K**_**d**_ **± SE (nM)**
MT-AV	0.572 ± 0.281
PNP-AV	0.406 ± 0.108
CD-AV	0.061 ± 0.026

### Enzyme prodrug cytotoxic efficacy

We evaluated the cytotoxic effect of each enzyme prodrug therapy on PC-3 cells by comparing the cell viability on days 2, 4, and 6 (MT-AV and PNP-AV) or days 3, 6, and 9 (CD-AV) to day 0 on a per well basis, and results are presented as percent viability compared to day 0. Statistical significance was established by comparing cells treated with varying concentrations of prodrug (or drug analog, if available) to their corresponding control groups treated with 0 μM drug/prodrug on the same day (#, *p* < 0.05; *, *p* < 0.01; and **, *p* < 0.001). Additionally, cells treated with 50 pm docetaxel were compared to cells not treated with docetaxel at the same concentrations of prodrug/drug on the same day (^, *p* < 0.05; +, *p* < 0.01; and ++, *p* < 0.001).

**Figure 2:**
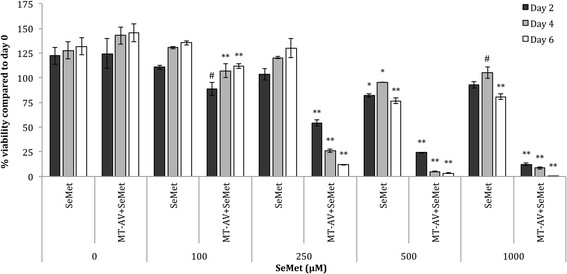
**Effect of SeMet conversion by MT-AV on PC-3 cell viability.** Cells treated with varying concentrations of SeMet were compared their corresponding control groups treated with 0 nM concentrations on the same day, and significant differences are denoted by # (*p* < 0.05), * (*p* < 0.01), and ** (*p* < 0.001). Data presented as mean ± SE (n = 3).

The cytotoxic effect of SeMet conversion by MT-AV was evaluated over 6 days with SeMet concentrations ranging from 0 to 1000 μM with 50 pM docetaxel (data not shown) and without docetaxel (Figure 2). MT-AV/SeMet treatment caused significant cytotoxicity starting at 250 μM SeMet, resulting in 64% viability by day 2 and 14% viability by day 6, with no growth inhibition for SeMet alone. At SeMet concentrations above 250 μM, MT-AV/SeMet killing velocity increased and near complete killing was achieved by day 6, with only slight growth inhibitory effects of SeMet alone. The addition of docetaxel treatment created no significant additional decreases in cell viability.

**Figure 3:**
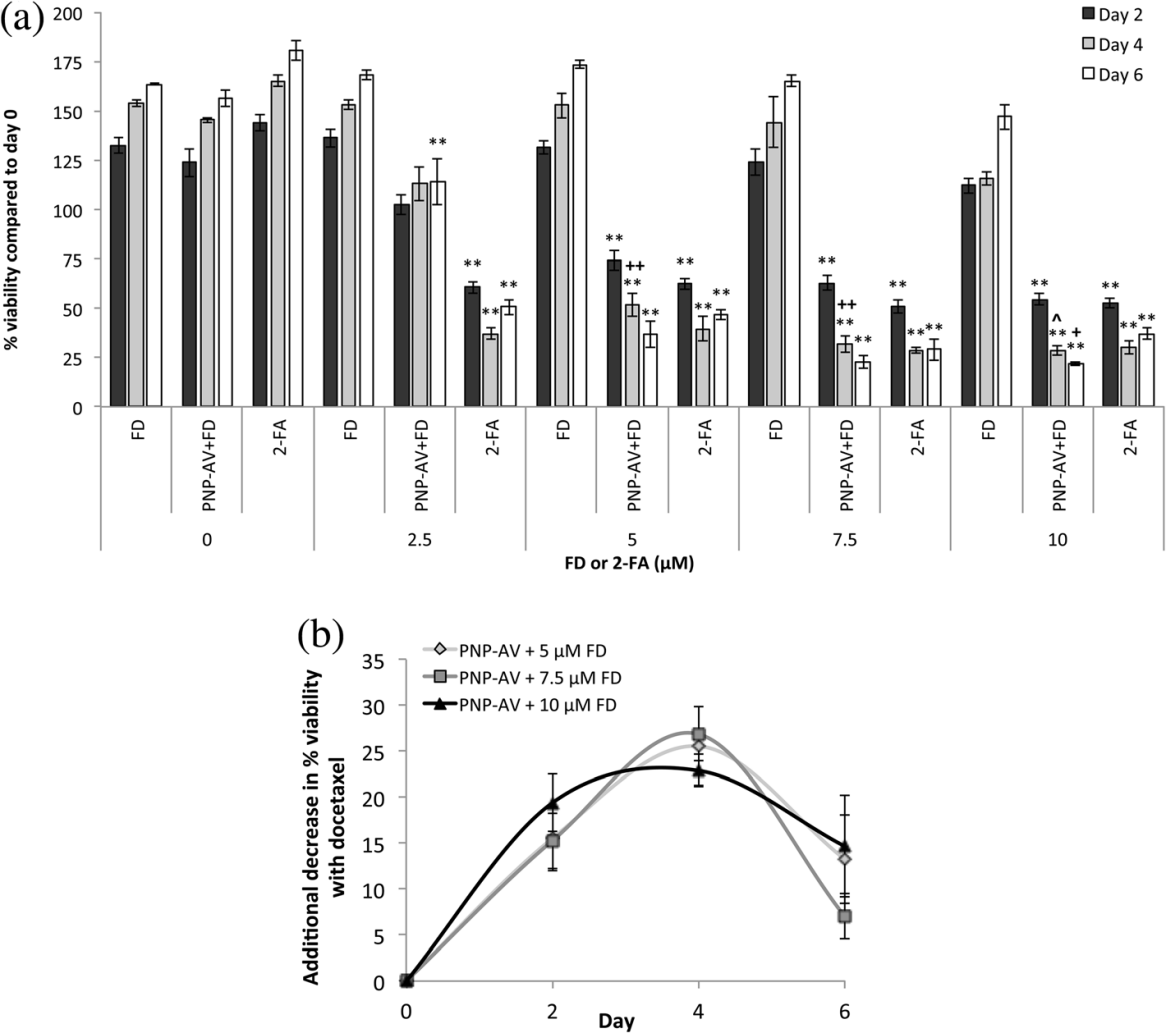
**Effect of FD conversion by PNP-AV with 50 pM docetaxel on PC-3 cell viability. (a)** Cells treated with varying concentrations of FD or 2-FA were compared to their corresponding control groups treated with 0 nM concentrations on the same day, and significant differences are denoted by # (*p* < 0.05), * (*p* < 0.01), and ** (*p* < < 0.001). Cells treated with 50 pm docetaxel (shown) were compared to cells not treated with docetaxel (see Additional file 1: Figure S1) at the same concentrations of FD or 2-FA on the same day, and significant differences are denoted by ^ (*p* < 0.05), + (*p* < 0.01), and ++ (*p* < 0.001). Data presented as mean ± SE (n = 3). **(b)** Additional decreases in cell viability afforded by the addition of 50 pM docetaxel to the PNP-AV system efficacy, shown for prodrug titers for which docetaxel treatment influenced treatment outcome. Results shown as non-docetaxel treated % viability minus docetaxel treated % viability to obtain a measure of additional cell killing with docetaxel treatment that alone has no significant effect on cell growth. Data presented as mean ± SE (n = 6).

The cytotoxic effect of 2-FA converted from FD by PNP-AV in the presence (Figure 3(a)) and absence (see Additional file 1: Figure S1) of docetaxel treatment was determined over 6 days with FD or 2-FA concentrations ranging from 0 to 10 μM. PNP-AV in combination with 5 μM FD was the lowest concentration of prodrug that showed significant cytotoxic effects, reaching 37% viability by day 6 with docetaxel treatment and 50% viability without docetaxel treatment. Cytotoxicity effects increased with increasing FD concentration up to 10 μM, reaching 22% viability by day 6 with docetaxel treatment and 37% viability without docetaxel treatment. Treatment with PNP-AV/FD was statistically indistinguishable from 2-FA treatment alone at concentrations ≥ 5 μM for docetaxel treated cells, but for non-docetaxel treated cells this treatment similarity did not occur at the concentrations evaluated (see Additional file 1: Figure S1). FD treatment alone did not show any cytotoxic effects at concentrations ≤ 10 μM. Treatment only with docetaxel did not affect PC-3 cells, but the addition of docetaxel significantly enhanced PNP-AV/FD cytotoxic efficacy at 5 μM FD concentrations and above as indicated (^, +, ++) in Figure 3(a). Additional decreases in % cell viability that occurred with docetaxel treatment are presented in Figure 3(b) for FD concentrations of 5, 7.5, and 10 μM. On day 2, additional decreases in % cell viability ranged from 15-19%. The highest additional cytotoxicity occurred on day 4 (22-27%), and the effect diminished by day 6.

**Figure 4:**
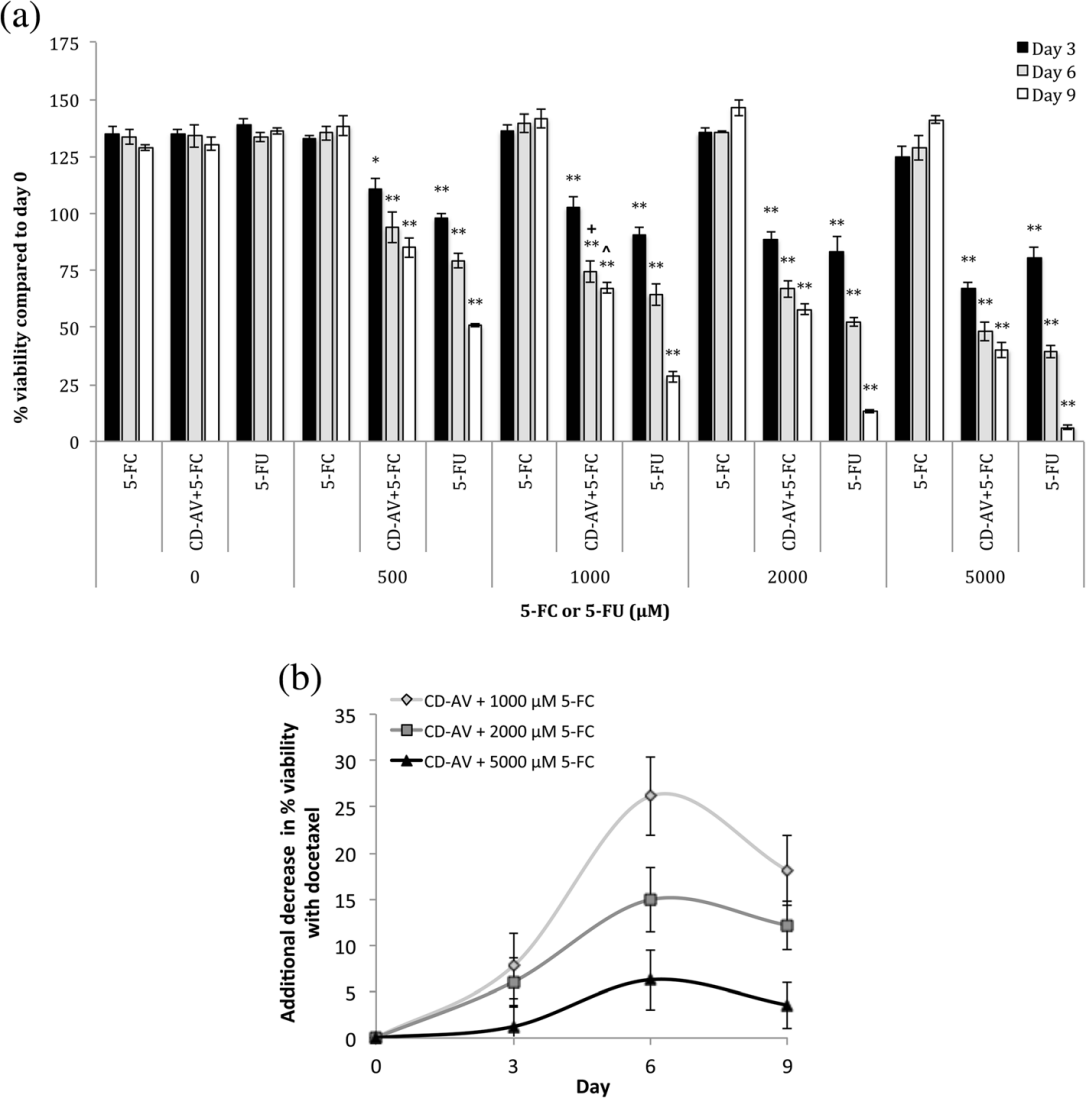
**Effect of CD-AV conversion of 5-FC with 50 pM docetaxel treatment on PC-3 cell viability. (a)** Cells treated with varying concentrations of 5-FC or 5-FU were compared their corresponding control groups treated with 0 nM concentrations on the same day, and significant differences are denoted by # (*p* < 0.05), * (*p* < 0.01), and ** (*p* < 0.001). Cells treated with 50 pm docetaxel were compared to cells not treated with docetaxel (see Additional file 1: Figure S2) at the same concentrations of 5-FC or 5-FU on the same day and significant differences are denoted by ^ (*p* < 0.05) or + (*p* < 0.01). Data presented as mean ± SE (n = 3). **(b)** Additional decreases in cell viability by the addition of 50 pM docetaxel, which alone has no effect on cell viability, for prodrug concentrations where docetaxel additional affected treatment outcomes. Results shown as non-docetaxel treated % viability minus docetaxel treated % viability to obtain a measure of additional cell killing with docetaxel treatment that alone has no significant effect on cell growth. Data presented as mean ± SE (n = 6).

The cytotoxic effect of 5-FU converted from 5-FC by CD-AV with (Figure 4(a)) and without (see Additional file 1: Figure S2) docetaxel treatment was evaluated over 9 days with concentrations of 5-FC/5-FU ranging from 0 to 5000 μM. CD-AV/5-FC treatment caused significant cytotoxicity at all concentrations above 500 μM but was most effective at 5000 μM 5-FC resulting in 40% viability by day 9 with docetaxel treatment and 44% without docetaxel treatment. No significant increases in cytotoxicity occurred past 5000 μM CD-AV/5-FC or 5-FU treatment (data not shown). 5-FC treatment alone exhibited no cytotoxic effect for both docetaxel and non-docetaxel treated cells. Treatment with the drug analog 5-FU showed significantly more cytotoxic effects than treatment with CD-AV/5-FC from day 6 onwards and resulted in ~6% viability for both docetaxel and non/docetaxel groups. Treatment only with docetaxel had no effect on PC-3 cells, but the addition of docetaxel significantly affected the killing efficacy of the CD-AV/5-FC system as indicated in Figure 4(a) (^, +). The additional decreases in % viability as a result of docetaxel addition are presented in Figure 4(b). Docetaxel affected CD-AV/5-FC efficacy in an inverse dose dependent manner, with respect to the prodrug, as the largest additional decreases in % viability consistently occurred at 1000 μM 5-FC and the smallest additional decreases were consistently seen at 5000 μM 5-FC. As for PNP-AV/FD, the impact of docetaxel was greatest in the middle of the study reaching an additional decrease in % viability of 26% on Day 6 for 1000 μM 5-FC.

## Discussion

The MT-AV/SeMet enzyme prodrug system emerged as a promising treatment option as it displayed significant cytotoxicity *in vitro* at feasible *in vivo* SeMet treatment concentrations. The median lethal dose (LD_50_) of SeMet *in vivo* for female nude mice is 12.5 mg/kg [41],[42], which translates to ~1100 μM *in vitro*. A high degree of prostate cancer cell killing was achieved with SeMet concentrations as low as 250 μM with minimal cytotoxicity of SeMet alone, suggesting a feasible window of opportunity for *in vivo* treatment translation.

PNP-AV also emerged as a feasible option for treating PC as it displayed high killing velocity and killing efficacy, both of which are important for clinical translation. PNP-AV also showed the most robust increase in cell killing efficacy in the presence of docetaxel. PNP-AV/FD (with docetaxel) created up to 78% cytotoxicity over 6 days at an FD concentration of 10 μM *in vitro*, which translates to less than 0.1% of the LD_50_ (~1200 mg/kg) for FD in female nude mice, indicating that this therapy could be administered with minimal harm to healthy tissues.

CD-AV/5-FC treatment was not as effective or as rapid as MT-AV/SeMet or PNP-AV/FD treatment, and we therefore conclude that CD-AV/5-FC would most likely not be effective *in vivo*. Additionally, the prodrug concentration necessary to elicit a cytotoxic effect was significantly higher than for the MT-AV and PNP-AV systems, although even at the highest level of prodrug, there was no effect of the prodrug by itself.

All three FPs exhibited relatively strong binding to PS on PC-3 cells with dissociation constants less than previously reported dissociation constants for AV alone to PS (2.7–15.5 nM) [43],[44]. The multimeric structure of each FP likely allows for multiple AV to PS bonds per FP, and we believe this contributes to the observed strong binding of FPs to PS.

Subtoxic docetaxel treatment significantly but selectively increased the cytotoxic efficacy of our enzyme prodrug systems, suggesting that at least two of our FP/prodrug combinations are sensitive to the extent of PS outer leaflet exposure. Tumor xenografts in murine models expose ~ 35% of PS on the external leaflet [18] (with > 10^6^ PS molecules per cell [22]), but docetaxel treatment can increase PS expose and thereby the number of potential FP binging sites by up to 70% [22], effectively doubling the FP targets and thereby creating quicker and more powerful treatment. The additional decrease in cell viability caused by introducing docetaxel to the PNP-AV and CD-AV enzyme prodrug treatments is seen in Figures 3(b) and 4(b), respectively. The maximum effect was present at about the midpoint of each study, i.e. at day 4 in the PNP-AV system and at day 6 in the CD-AV system. We believe that the peak in this effect is a result of increased initial prodrug to drug turnover enabled by the increased presence of bound FP’s due to the additional availability of PS binding sites. Therefore, the addition of docetaxel causes the enzyme prodrug treatment to speed up initially; and later as the number of viable cells dwindles, the effect becomes relatively less noticeable.

We employed subtoxic treatment levels of docetaxel, as we were interested in the PS exposure effects of docetaxel and not its cytotoxic capabilities. Not only did docetaxel treatment alone have no growth inhibitory or cytotoxic effects, but the addition of docetaxel treatment did not alter the cytotoxic efficacy of the drug analogs, 2-FA and 5-FU. This indicates there was no synergism present between the drugs generated by our enzyme prodrug therapies and docetaxel. Therefore, it is probable that the increased cytotoxic effect afforded by docetaxel treatment was in fact due to an increase in PS exposure providing an increase in available binding sites for our FPs.

Unexpectedly, docetaxel treatment did not increase MT-AV cytotoxicity on PC-3 cells. We propose that this effect did not occur because the killing efficacy of the MT-AV system may already be saturated at feasible SeMet concentrations without docetaxel. Saturation could arise if the amount of MT-AV able to bind without docetaxel treatment is sufficient to convert the available SeMet, as any additional MT-AV binding would increase the initial SeMet turnover rate but would not ultimately affect the quantity of reactive oxygen species the cells are exposed to.

Further validation of the MT-AV and PNP-AV systems will consist of *in vivo* work in murine xenograft models. The immunogenicity of the FP systems can be addressed via functionalization of human homologs [45],[46] or via PEGylation [47].

## Conclusions

In conclusion, we have substantiated the feasibility of two, novel, non-invasive treatments for prostate cancer and its metastases with minimal threat to healthy tissues. We were able to achieve both tight binding, with dissociation constants in the low nanomolar range, and excellent cytotoxic efficacy for the MT-AV and PNP-AV enzyme prodrug systems. Additionally we have shown the utility of subtoxic docetaxel treatment for increasing the cytotoxic potential of annexin V-targeted enzyme prodrug systems.

## Abbreviations

FP: Fusion protein

AV: Annexin V

MT: L-methioninase

PNP: Purine nucleoside phosphorylase

CD: Cytosine deaminase

PC: Prostate cancer

ADPET: Antibody-directed enzyme prodrug therapy

GDEPT: Gene-directed enzyme prodrug therapy

VDEPT: Viral-directed enzyme prodrug therapy

PS: Phosphatidylserine

FD: Fludarabine

5-FC: 5-fluorocytosine

SeMet: L-selenomethionine

HAAE-1: Human abdominal aorta endothelial cells

K_d_: Dissociation constant

LD_50_: Median lethal dose

## Competing interests

The only potential competing interest we declare is an application for a U.S. patent on this targeted enzyme prodrug therapy system.

## Authors’ contributions

KPG, CK, and RGH designed the experiments. KPG conducted experiments and analyzed the data. KPG and RGH wrote the manuscript. All authors read and approved the final manuscript.

## Additional file

## Supplementary Material

Additional file 1: **Figure S1.** Effect of FD conversion by PNP-AV on PC-3 cell viability. **Figure S2.** Effect of 5-FC conversion by CD-AV on PC-3 cell viability. Click here for file
