# Supplementary material for: Targeted enzyme prodrug therapy for metastatic prostate cancer – a comparative study of L-methioninase, purine nucleoside phosphorylase, and cytosine deaminase
Source: J Biomed Sci. 2014 Jul 22;21(1):65. doi: 10.1186/s12929-014-0065-3 (PMC4223417; doi:10.1186/s12929-014-0065-3)
Supplement: Additional file 1: — Figure S1. Effect of FD conversion by PNP-AV on PC-3 cell viability. Figure S2. Effect of 5-FC conversion by CD-AV on PC-3 cell viability. [file s12929-014-0065-3-S1.docx]

**Additional File 1: Figure S1**

**Additional File 1: Figure S1. Effect of FD conversion by PNP-AV on PC-3 cell viability.** Cells treated with varying concentrations of FD or 2-FA were compared their corresponding control groups treated with 0 nM concentrations on the same day, and significant differences are denoted by # (*p* < 0.05), * (*p* < 0.01), and ** (*p* << 0.001). Data presented as mean ± SE (n = 3).

**Figure 2 Effect of 5-FC conversion by CD-AV on PC-3 cell viability.** Cells treated with varying concentrations of 5-FC or 5-FU were compared their corresponding control groups treated with 0 nM concentrations on the same day, and significant differences are denoted by # (*p* < 0.05), * (*p* < 0.01), and ** (*p* < 0.001). Data presented as mean ± SE (n = 3).
